# Clinical-functional correlation with brain volumetry in severe perinatal asphyxia: a case report

**DOI:** 10.1186/s13052-024-01633-w

**Published:** 2024-04-09

**Authors:** Juan Pablo Velasquez-Minoli, Natalia Cardona-Ramirez, Hernan Felipe Garcia-Arias, Feliza Restrepo-Restrepo, Gloria Liliana Porras-Hurtado

**Affiliations:** 1 Salud Comfamiliar, Caja de Compensación Familiar de Risaralda, Pereira, Colombia; 2 Automatic Research Group, Universidad Tecnológica de Pereira, Pereira, Colombia; 3 Investigación y Docencia, Hospital Pablo Tobón Uribe, Medellín, Colombia; 4 pereira, Colombia; 5 SISTEMIC Research Group, Universidad de Antioquia, Medellín, Colombia

**Keywords:** Case report, Hypoxic-ischemic encephalopathy, Perinatal asphyxia, Pathophysiology, Neurodevelopmental, Machine learning, 3D Volumetric model

## Abstract

**Background:**

Hypoxic-ischemic encephalopathy (HIE) appears in neurological conditions where some brain areas are likely to be injured, such as deep grey matter, basal ganglia area, and white matter subcortical periventricular áreas. Moreover, modeling these brain areas in a newborn is challenging due to significant variability in the intensities associated with HIE conditions. This paper aims to evaluate functional measurements and 3D machine learning models of a given HIE case by correlating the affected brain areas with the pathophysiology and clinical neurodevelopmental.

**Case presentation:**

A comprehensive analysis of a term infant with perinatal asphyxia using longitudinal 3D brain information from Machine Learning Models is presented. The clinical analysis revealed the perinatal asphyxia diagnosis with APGAR <5 at 5 and 10 minutes, umbilical arterial pH of 7.0 BE of -21.2 mmol / L), neonatal seizures, and invasive ventilation mechanics. Therapeutic interventions: physical, occupational, and language neurodevelopmental therapies. Epilepsy treatment: vagus nerve stimulation, levetiracetam, and phenobarbital. Furthermore, the 3D analysis showed how the volume decreases due to age, exhibiting an increasing asymmetry between hemispheres. The results of the basal ganglia area showed that thalamus asymmetry, caudate, and putamen increase over time while globus pallidus decreases. Clinical outcomes: spastic cerebral palsy, microcephaly, treatment-refractory epilepsy.

**Conclusions:**

Slight changes in the basal ganglia and cerebellum require 3D volumetry for detection, as standard MRI examinations cannot fully reveal their complex shape variations. Quantifying these subtle neurodevelopmental changes helps in understanding their clinical implications. Besides, neurophysiological evaluations can boost neuroplasticity in children with neurological sequelae by stimulating new neuronal connections.

**Supplementary Information:**

The online version contains supplementary material available at 10.1186/s13052-024-01633-w.

## Background

Hypoxic-ischemic encephalopathy (HIE) affects brain areas with higher metabolic rates and active myelination processes [1]. HIE is associated with low neurodevelopmental outcomes and mortality rates up to $6\%$ [2–6]. In severe asphyxia, frequent sequelae such as the motor deficit, cerebral palsy, and sensory and cognitive abnormalities are estimated [7, 8].

In term newborns, some brain areas are likely to be injured, such as deep grey matter (thalamus and lateral geniculate nuclei, putamen, hippocampus, dorsal and lateral brainstem) and the white matter subcortical periventricular áreas. Furthermore, the cerebral cortex can also be affected in severe and prolonged ischemia, causing a critical prognosis [1]. Clinical conditions in children with HIE have been related to specific imaging findings through different techniques. In recent years, the most suitable technique is Magnetic Resonance Imaging (MRI). MRI allows the evaluation of different brain tissues and lesion distribution. This technique is the gold standard for predicting adverse clinical results with high specificity and sensitivity [9]. Volumetric MRI allows a schematic evaluation of the depth of lesions in vivo by visualizing changes in the volume of the structures for follow-up over time [10–12].

Moreover, this type of structural image shows changes in the brain by quantifying and reconstructing each area of interest (i.e., basal ganglia area in HIE) [13]. In medical imaging, machine learning models, such as deformable models [14], allow reconstructing the contour associated with each brain structure using 3D interpolation methodologies. Therefore, a 3D anthropometric model of both the injury and the contour related to each brain structure will allow health professionals to determine the injury factor and thus monitor the brain volume’s deformation (i.e., changes in the brain shape) over time. Besides, this condition leads to motor and cognitive alterations of variable severity, including cerebral palsy, seizures, spasticity, attention deficit, hyperactivity, mental retardation, and other neuropsychiatric syndromes with delayed clinical onset.

In pediatrics, psychomotor development is evaluated using scales such as the Bayley scale, Battelle Developmental Inventory-Jean Newborg scale, The Psychomotor Development Test (TEPSI), The National Research Test for Child Development, and the Abbreviated Development Scale. The standardized Bayley Scale allows for evaluating children’s mental, psychomotor, and behavioral development between 1 and 42 months. Besides, it has scientific support through numerous international publications and is considered one of the leading global tools for assessing child development. Therefore, a prospective study on infants with HIE has been performed in central-western Colombia for the last three years. This study seeks to clinically correlate the functional outcome with the Bayley scale and neurodevelopmental in perinatal asphyxia by incorporating volumetric models to quantify the clinical outcome through a multidimensional diagnosis with functional measurement tools and machine learning techniques.

This paper shows how functional measurements and 3D machine learning models allow a relevant evaluation of a given HIE case by correlating the affected brain areas with the pathophysiology, clinical neurodevelopmental, and mechanisms of neuronal injury.

## Case presentation

### Clinical and functional analysis

The patient, a male born at 39 weeks of gestational age, underwent a cesarean section delivery due to prolonged labor and acute fetal distress. At birth, he weighed 2.5 kg and measured 51 cm in height. Clinical evaluation yielded a diagnosis of perinatal asphyxia, characterized by hypotonia, cyanosis, bradycardia, and a lack of respiratory effort, leading to APGAR scores below five at 5 and 10 minutes. Further analysis revealed an umbilical arterial pH 7.0 with a base excess (BE) of -21.2 mmol/L. The presence of neonatal seizures was confirmed through EEG, necessitating invasive mechanical ventilation.

Within the first month of life, the patient was diagnosed with SARNAT grade II-III encephalopathy, early-onset sepsis, and right apical atelectasis. Additional EEG assessments confirmed ongoing seizures, while a pediatric echocardiogram indicated normal cardiac function. A transfontanellar ultrasound performed on the third day of life revealed a loss of differentiation between gray and white matter in the lenticular nuclei and thalamus without evidence of hemorrhagic collections. These findings pointed to the involvement of the basal ganglia and thalamus.

Hypothermia therapy, a recommended treatment, was not administered immediately at birth due to limited availability in Colombian hospitals. Subsequently, a comprehensive review of the therapy implemented, a detailed assessment of epilepsy progression using continuous EEG videotelemetry, and a functional analysis employing the Bayley Child Development Assessment Scales-III (BSID-III) at 1, 18, and 39 months of age were undertaken. The primary objective of these assessments was to effectively quantify the patient’s neurodevelopmental progress, covering aspects related to cognitive, motor, and language development. These evaluations play a pivotal role in understanding the patient’s condition and guiding the course of treatment and support throughout his early years.

### Structural analysis

MRI sequences T1, T2, and DWI were captured from a Siemens Healthineers 1.5*T* with a volumetric MPRAGE $1mm\times 1mm \times 1mm$ voxel size. Diffusion sequences on resonance are evaluated by the presence of areas of cytotoxic edema given by hyperintensity in the *b*1000 sequences and hypointensity in the ADC map. In the other sequences, the brain anatomy is evaluated, observing the presence of edema of the white matter, loss of differentiation between the white and grey matter, and congenital malformations or other alterations. The appearance of encephalomalacia and gliosis areas was evaluated at one year and three years. The affected areas’ volume loss is identified with increased subarachnoid space and surrounding hyperintensity in FLAIR sequences.

### 3D volumetric model

**Fig. 1:**
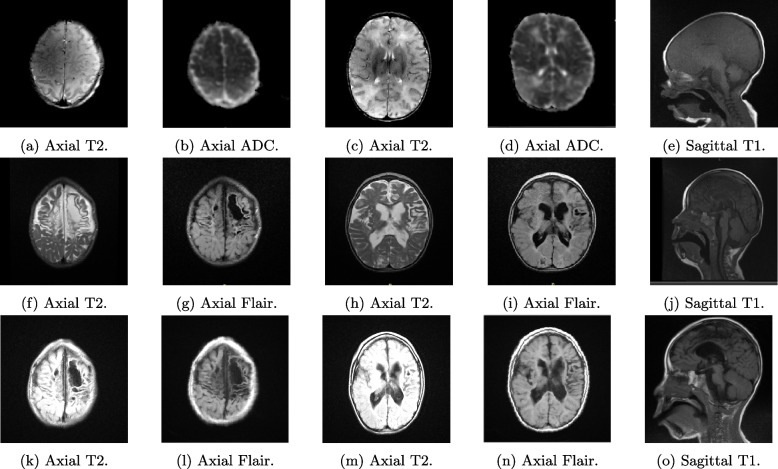
**Row 1:** Initial magnetic resonance. **a** and **b** Axial sections T2 and ADC at the level of the semioval centers. **c** and **d** Axial T2 and ADC sections in nucleus basal regions. **e** Parasagittal T1 image. **Row 2:** Control magnetic resonance, 18 months later. **f** and **g** Axial sections T2 and Flair at the level of the semioval centers. **h** and **i** Axial T2 and Flair sections in nucleus basal regions. **j** Parasagittal T1 image. **Row 3:** Control magnetic resonance, 39 months later. **k** and **l** Axial sections T2 and Flair at the level of the semioval centers. **m** and **n** Axial T2 and Flair sections in nucleus basal regions. **o** Parasagittal T1 image

A senior pediatric neuroradiologist labeled all MRI volumes for macro structures and lesions at each recording time to evaluate our volumetric model. Thus, ground-truth labels were made on 3DSlicer software. A brain volumetric analysis is presented based on unsupervised machine learning models to learn the shape structure at different times. Besides, the MRI volumes were processed using Infant FreeSurfer software, which allows automatic and complete segmentation of the cortical and subcortical areas of the brain. As a result, we obtain a label map that can be evaluated and used to extract areas of interest associated with brain structures [15, 16]. Finally, we perform a Marching Cubes model to obtain the 3D shapes of the brain structures and the affected regions. To this end, a brain surface is reconstructed by deformable contours from a three-dimensional discrete scalar field, which refers to a magnetic resonance image [17] (Fig. 1).

### Clinical-functional temporal outcomes

Table 1 shows a temporal analysis of a patient’s medical outcome at three distinct time intervals: the first month, 18 months, and 39 months of life. This table correlates the clinical features with magnetic resonance imaging (MRI) findings, diagnostic tests, and developmental milestones. This combined information gathering helps us understand how the patient’s condition changes, emphasizing the evolving clinical context.

In the first month of life, the patient exhibited neurological abnormalities, such as axial and appendicular hypotonia, and the absence of several reflexes. An MRI at 17 days revealed extensive areas of diffusion restriction in various brain regions. Despite extensive genetic and metabolic studies, the cause remained elusive, leading to a diagnosis of classic perinatal asphyxia. The Bayley scale at one month indicated severe cognitive and motor delays, and the patient experienced the first seizure shortly after birth. Treatment for drug-resistant epilepsy included vagus nerve stimulation, levetiracetam, and phenobarbital. Neurodevelopmental therapies commenced at month 12.

At 18 months, the patient showed improvement, with increased interaction and attention, but continued motor and cognitive deficits. MRI images revealed multicystic encephalomalacia and volume loss in specific brain regions. By 39 months of age, the patient had developed a good level of interaction and empathy. However, motor and cognitive development remained significantly delayed, with further evidence of brain atrophy, including multicystic encephalomalacia and subcortical cysts in the brain’s frontal regions. The patient’s condition showed limited improvement over the first 39 months of life, with persistent cognitive and motor challenges and ongoing structural brain abnormalities.

**Table 1:** Summary of Patient’s Condition at Different Stages

Age	Clinical Features	MRI Findings
1 Month	Neurological evaluation showed axial and appendicular hypotonia, absent reflexes, and extensive areas of diffusion restriction in frontal, occipital, and anterior temporal regions. Suspected inherited metabolic disease.	Cytotoxic edema in frontal lobes, loss of differentiation between white and gray matter, and alterations in basal nucleus regions (Fig. 1a-d).
	**Clinical Therapies:** Topiramate, clonazepam.	
	**EEG results:** At two months, multifocal epileptiform activity with alternating sharp and delta waves and a burst suppression pattern occurred predominantly in the bilateral parasagittal frontal region.	
	**Diagnostic Tests:** Metabolic study, genetics study, Bayley scale	
	**Bayley Scale Results:** Cognitive 0.1 percentile, Language $< 0.1$ percentile	
18 Months	Patient presented with esotropia, axial dystonia, spastic hemiparesis predominantly on the right, and continued cognitive and motor impairment.	Extensive multicystic encephalomalacia in semioval centers, volume loss in insula lobes and occipital poles, and thinning of the corpus callosum (Fig. 1f-h).
	**Clinical Therapies:** Phenobarbital, vigabatrin, lacosamide, levetiracetam.	
	**EEG results:** Continuous 24-hour video telemetry revealed asymmetrical slow theta/delta waves, primarily in the right cerebral hemisphere. Burst-suppression pattern with spike-and-slow-wave complexes and frequent epileptiform activity in the left posterior and front-central regions.	
	**Bayley Scale Results:** Cognitive 2 months 10 days, Expressive Language 2 months 20 days	
39 Months	Patient had axial hypotonia, spastic quadriparesis, generalized hyperreflexia, and limited hip abduction. Guttural sounds and low comprehension language.	Evidence of cortical and periventricular volume loss, multicystic encephalomalacia with subcortical cysts, and subcortical cysts in the frontal lobes (Fig. 1k-o).
	**Clinical Therapies:** Phenobarbital, vigabatrin, lacosamide, levetiracetam, vagal stimulation therapy, initiation of physical, functional, and speech therapy.	
	**EEG results:** Continuous 24-hour video-EEG telemetry reveals frequent interictal epileptiform activity in spikes, poly-spikes, and high-voltage sharp waves. This activity was asynchronous, independent, and primarily localized in the bilateral temporo-parieto-occipital regions with a left occipital predominance..	
	**Bayley Scale Results:** Cognitive 2 months 10 days, Expressive Language 2 months 20 days	

### Brain volumetry

**Fig. 2:**
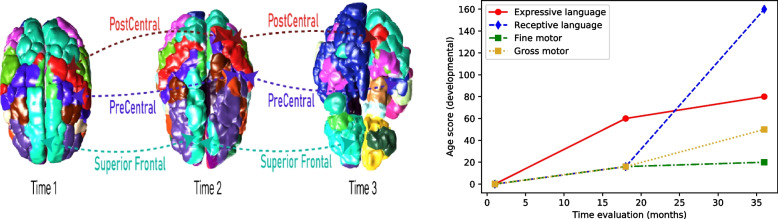
Brain volumetry and Bayley score at 3 different times

**Fig. 3:**
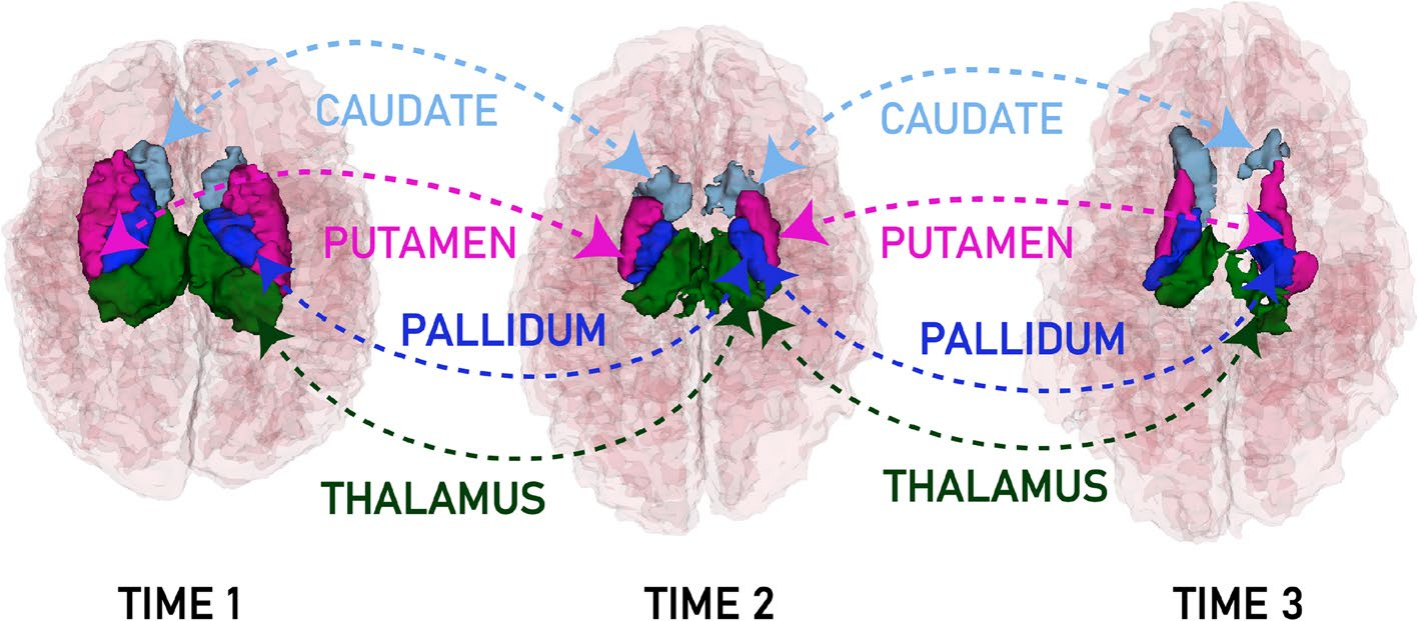
Basal ganglia in 3 different times

The automatic parceling allows us to compute macro and microstructures to correlate the neurodevelopmental among time with the Bayley scale. Figures 2 and 3 show the 3D brain reconstruction for the brain cortex and basal ganglia area, respectively.

In addition, Fig. 2 shows how the volumetric changes in the 3D model show changes that can be correlated with the Bayley scale scores for developmental age.

Automatic parceling also makes it conceivable to calculate the percentage of volume and percentage of asymmetry between hemispheres, as Table 2 shows.

**Table 2:** Macro and micro structures volume and asymmetry between both hemisphere at 3 different times. The volume percentage is based on the intracranial cavity for which is measured in $cm^3$ (time 1: 344.473, time 2: 635.510, time 3: 633.758)

Tissue	Time 1 ($cm^3/\%$)	Time 2 ($cm^3/\%$)	Time 3 ($cm^3/\%$)	Time 1 Asymmetry ($\%$)	Time 2 Asymmetry ($\%$)	Time 3 Asymmetry ($\%$)
**White Matter**	124.162(36.04)	124.630(19.61)	88.092(13.90)	8.61	18.23	42.36
**Gray Matter**	159.474(46.29)	240.388(37.82)	201.972(31.86)	10.924	4.770	27.647
**Brain**	283.639(82.34)	365.024(57.43)	290.084(45.77)	9.912	9.367	32.118
**Thalamus**	10.510(3.05)	52.493(0.82)	22.182(0.35)	2.159	5.497	65.293
**Caudate**	3.552(1.03)	15.760(0.24)	20.914(0.33)	28.461	34.742	141.981
**Putamen**	6.583(1.91)	37.749(0.59)	28.963(0.45)	15.409	23.137	24.889
**Globus Pallidus**	3.111(0.90)	16.587(0.26)	19.076(0.30)	30.404	11.607	4.325

Table 2 shows how the volume decreases in reason of age, also exhibiting an increasing asymmetry between hemispheres. Besides, for the basal ganglia area, we can see that the asymmetry of the thalamus, caudate, and putamen increases over time, while globus pallidus decreases. This behavior states that the cognitive relation with the emotional elicitations allows the memory controller through stimuli.

To assess the correlation between the patient’s outcome and the size of the brain structures in a term infant affected by Perinatal Asphyxia (HIE), we compare the thalamic size of the affected patient with that of a non-affected term infant. Additionally, we analyze this comparison in the context of the basal nuclei (basal ganglia) sizes presented in the Table 2. Table 3 shows some average values for brain structure sizes for a non-affected term infant. By comparing the thalamic size of the HIE-affected patient with that of a non-affected term infant, we observe that the thalamus in the affected patient at time 1 ($10.51 cm^3$) is slightly larger than the thalamus in the non-affected term infant ($9.12 cm^3$). This is an interesting finding, as it suggests that the thalamic volume in the HIE-affected patient may not be the primary factor responsible for the condition. Instead, other structures within the basal ganglia, such as the caudate and putamen, show significant size differences in the HIE-affected patient, potentially playing a more substantial role in the patient’s outcome. Furthermore, the discrepancy between the thalamic size and the size of other basal nuclei emphasizes the complexity of HIE and its impact on different brain regions.

**Table 3:** Average brain volumes in a non-affected term infant [18]

Structure	Volume (cm^3^)
Cerebral White Matter	$146.61 \pm 16.44$
Cortical Gray Matter	$119.79 \pm 16.08$
Brain	$316.82 \pm 35.52$
Thalamus	$9.12 \pm 1.22$
Hippocampi	$1.52 \pm 0.25$

**Fig. 4:**
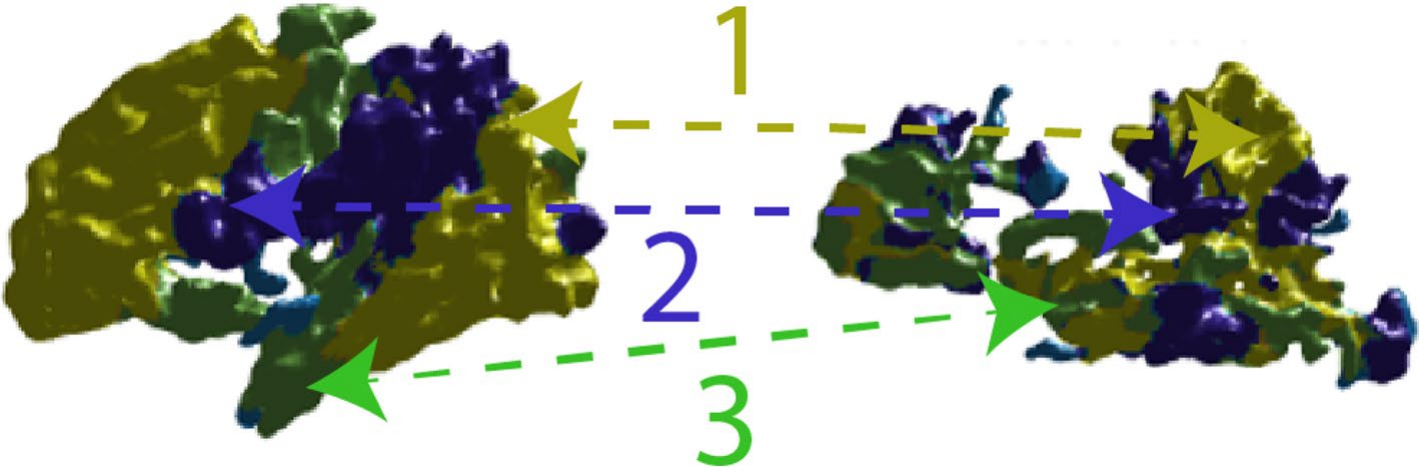
Result of unsupervised learning model in the right hemisphere (time 1 vs. time 3)

The Fig. 4 shows the results obtained by training an automatic correspondence model. In this case, to determine the volumetric changes of neurodevelopment. The model finds areas that meet a similar behavior by examining the same curvature in detail.

Figure 4 also shows a significant result found by the model. In this case, it is possible to observe how different zones that occupied a large part of the surface have been strongly reduced at three. Besides, by looking at zones one and two in the figure, we can see that volume was reduced significantly, while the third zone expanded, occupying spaces where it was not present before. This behavior correlates with the patient’s functional state in which, through personalized neurostimulation, some positive clinical outcomes are reached. As a result, we observed a relevant level of relationship with his environment empathy and performed emotional communications (i.e., recognizing faces and emotions).

## Discussion and conclusions

According to the affected brain areas, we found variable degrees of clinical compromise related to the pathophysiology and the mechanisms of neuronal injury. Besides neuronal susceptibility, the severity of hypotension, duration of hypoxia, and gestational age are leading factors that influence the clinical outcome. For example, limb lesions, basal cells, and thalamus are associated with more significant motor impairment, spastic cerebral palsy, and epilepsy [7]. Besides, cerebral cortex lesions can cause language and other higher motor and cognitive alterations. Thus, periventricular or subcortical white matter lesions are associated with cognitive and behavioral alterations [2].

In this case, the combination of deep grey and white matter injury is the most severe injury pattern. Cortical injury, i.e., cortical necrosis, is very often the result of prolonged seizures and secondary to the hypoxic-ischemic insult. An alteration of the sensory capacities appears as a consequence of cortical lesions predominantly in the vision system [19]. The case presented in this manuscript is typical of severe asphyxia with significant motor impairment, spastic cerebral palsy, and epilepsy. The described case shows no congenital malformations, metabolic disorders, chromosomal diseases, or genetic diseases. Besides, we observed an increase in myelinated areas and a decrease in the posterior part of the internal capsule of the brain.

It is important to note that these neurological changes vary with neurodevelopmental maturity. However, redistribution of blood flow associated with hypoxia and hypotension prioritizes contributing to the most metabolically active brain areas. Thus, the three main patterns of brain damage described during HIE are strongly related to vascular immaturity, on which volumetric brain areas of parasagittal substance appear injured. Furthermore, the adjacent vascular territory’s watershed lesion borders vascular territories in a full-term newborn with the parasagittal cortex and subcortical white. Besides, the periventricular leukomalacia / Germinal matrix, intraventricular hemorrhage of different degrees, and the thalamic pattern involving the basal ganglia and perirolandic cortex [12]. Thus, the volumetric reconstruction of the structural MRI image allows for identifying a thalamic pattern in the case. From the volumetric measurements, we observed that functional evaluation of the Bailey scale correlates suitably with the neurodevelopmental. Hence, some brain regions related to motor areas are less developed than cognitive areas. The proposed unsupervised model finds the shape relations between brain structures over time. Hence, it is possible to evaluate neurodevelopmental changes in surface areas candidates for neuroplasticity.

It is worth noting that this is an initial case report on which volumetric analysis of neurodevelopmental was used to evaluate quantitatively subtle brain changes in a post-therapeutic intervention. It is worth noting that this is an initial case report on which volumetric analysis of neurodevelopmental was used to evaluate quantitatively subtle brain changes in a post-therapeutic intervention. However, these results are part of a large population study in which we aim to capture those correlation variables associated with better estimates for positive clinical outcomes.

The above case shows that it is expected to find a decrease in the parasagittal and subcortical white matter in mild to moderate encephalopathy. Also, it is easy to find edema areas due to loss of differentiation of the white matter and cortex in T2. Moreover, a progressive increase in the intensity of T1 in the first weeks joint a flattening with decreased cerebral cortex volume [7, 12]. Nevertheless, in severe or profound encephalopathy, it is common to find basal ganglia lesions, bilateral ventrolateral thalamus, with selective necrosis described as a “Basal ganglion-thalamus pattern” of the hippocampus brainstem and cerebral cortex. In addition, hyperintense signals on T2 in the first-hour progress to hypointensity at 7-10 days.

We found approximately 40-80%

Volumetric MRI has proven to be an essential tool for studying the clinical-radiological correlation of different patterns in secondary injury to perinatal asphyxia. Since it allows the quantitative assessment of even the most subtle effects on the volumes of brain structures and thus be able to determine the relationship with the clinical changes seen in patients as we state in this paper [20, 21]. In addition, since the structural MRI lacks information related to neurodevelopmental in times two and three (i.e., not found by the medical specialist), with the benefit of the machine learning model, we showed how unsupervised reconstruction of brain structures allows relevant quantification of structural neurological changes among time.

A recent study by Geva and collaborators evaluated the relationship of the decrease in the caudate nucleus volume in 20 patients with atrophy of the hippocampus secondary to HIE. They found that patients with a smaller volume of the caudate nucleus had lower performance in fine and coordinated movements [11]. Thus, these factors correlate the quantitative evaluation of the 3D volumetric model with the Bayley assessments. Therefore, the above patterns establish that white matter subcortical volumes were lower in children with lower results on the Bayle psychomotor development scale and a short statistical weight [22].

Several scientific articles have documented distinct MRI patterns of basal ganglia involvement in acquired disorders, such as cerebral palsy resulting from hypoxic-ischemic encephalopathy in full-term infants [23, 24]. It is important to note that conducting early MRI scans within the first eight days of life tends to underestimate basal ganglia injuries. Therefore, it is advisable to perform MRI scans at later stages for a more accurate assessment. In cases of early MRI, the visual evaluation of basal ganglia abnormalities proved less reliable (with $59\%$ reported as abnormal) than quantitative assessments (where $90\%$ were deemed abnormal, $p = 0.015$) [24, 25],. This discrepancy highlights the necessity for adopting quantitative assessment methods. Furthermore, a notably lower thalamus-to-basal ganglia Apparent Diffusion Coefficient (ADC) ratio was observed in infants subjected to hypothermia treatment.

In conclusion, we found that slight changes in the basal ganglia and cerebellum could only be seen by 3D volumetry. Hence, the robust quantification of neurodevelopmental where the shape of small structures such as the basal ganglia cannot be fully decoded from standard MRI examination. The quantitative assessment of even the most subtle effects on brain structures allows for determining the relationship with the clinical changes. Finally, strengthening specific stimulation through neurophysiological evaluations derives new neuronal connections to improve the neuroplasticity of children with neurological sequelae.

### Supplementary Information

**Additional file 1:** **Supplementary Table S1.** Comparison of brain volumetry changes at the three evaluation times.

## Data Availability

All data generated or analysed during this study are included in this published article and its supplementary information files.
