# Supplementary material for: Clinical-functional correlation with brain volumetry in severe perinatal asphyxia: a case report
Source: Ital J Pediatr. 2024 Apr 9;50:66. doi: 10.1186/s13052-024-01633-w (PMC11003057; doi:10.1186/s13052-024-01633-w)
Supplement: Supplementary file 1 — Additional file 1: Supplementary Table S1. Comparison of brain volumetry changes at the three evaluation times. [file 13052_2024_1633_MOESM1_ESM.pdf]

Reporte de Volumetría

Grupo de Investigación en Salud Comfamiliar

Volumen

Table 1: Información Básica

Paciente ID	Sexo	Edad	Fecha Resonancia
1	Masculino	17 Meses	14/07/2016

Table 2: Volumen Macro

Tissue	mm3	%
White Matter	124634,6594	19,61175341
Gray Matter	240389,742	37,82627053
Brain	365024,4014	57,43802394
Intracranial Volume	635510,0269	100

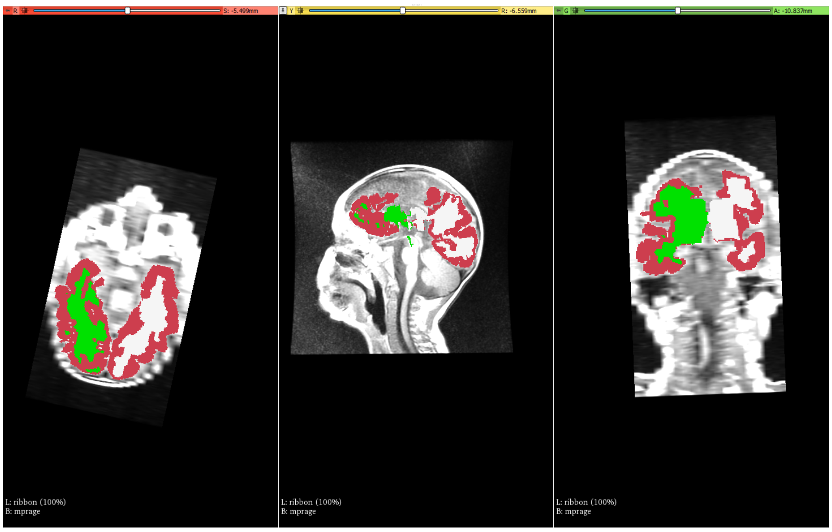

Figure 1: WM y GM

Table 3: Volumen Hemisferios

Tissue	Left (mm$\hat{3}$)	Right (mm$\hat{3}$)	Left (%)	Right (%)	Asymmetry (%)
**White Matter**	56636,25679	67998,40258	8,91193756	10,69981585	18,23272251
**Gray Matter**	117327,8683	123061,8737	18,46200113	19,3642694	4,770590716
**Total**	173964,1251	191060,2763	27,37393869	30,06408525	9,367127836

Table 4: Volumen Estructuras

structure_name	lf_volume (mm$\hat{3}$)	rh_volume (mm$\hat{3}$)	lf_volume (%)	rh_volume (%)	Asymmetry (%)
**bankssts**	569	1166	0,089534386	0,183474682	68,8184438
**caudalmiddlefrontal**	4644	2081	0,730751649	0,327453527	76,22304833
**cuneus**	1356	2496	0,213371928	0,392755408	59,19003115
**entorhinal**	776	1254	0,122106649	0,197321828	47,09359606
**fusiform**	3989	4784	0,627684825	0,752781199	18,1237889
**inferiorparietal**	5430	9851	0,854431837	1,55009356	57,86270532
**inferiortemporal**	5814	5516	0,914855746	0,867964275	5,260370697
**isthmuscingulate**	493	982	0,077575487	0,154521559	66,30508475
**lateraloccipital**	7842	9823	1,233969516	1,54568765	22,42853099
**lateralorbitofrontal**	4741	3786	0,74601498	0,595741977	22,39943708
**lingual**	2451	4423	0,385674481	0,69597643	57,37561827
**medialorbitofrontal**	6101	3008	0,960016324	0,47332062	67,91085739
**middletemporal**	4592	6593	0,722569245	1,037434458	35,78006258
**parahippocampal**	1098	933	0,172774615	0,146811216	16,24815362
**paracentral**	1598	2237	0,251451579	0,35200074	33,32464146
**parsopercularis**	3047	1612	0,479457423	0,253654534	61,60120197
**parsorbitalis**	1765	2060	0,277729686	0,324149095	15,4248366
**parstriangularis**	5227	1782	0,82248899	0,280404702	98,30218291
**pericalcarine**	1302	1141	0,204874816	0,179540834	13,18051576
**postcentral**	5670	5877	0,892196781	0,924769044	3,585346843
**posteriorcingulate**	627	649	0,098660914	0,1021227	3,448275862
**precentral**	4406	4967	0,693301414	0,781576968	11,97055372
**precuneus**	3457	4235	0,543972534	0,666393892	20,22880915
**rostralanteriorcingulate**	625	419	0,098346206	0,065931296	39,46360153
**rostralmiddlefrontal**	9336	6979	1,469056286	1,098173074	28,89365614
**superiorfrontal**	3644	10434	0,573397719	1,641830901	96,46256571
**superiorparietal**	9584	7097	1,508080061	1,116740838	29,81835621
**superiortemporal**	5432	6086	0,854746545	0,957656015	11,35613822
**supramarginal**	3849	5063	0,605655275	0,796682945	27,24416517
**frontalpole**	651	1598	0,102437408	0,251451579	84,21520676
**temporalpole**	2947	2339	0,46372203	0,368050841	23,00416194
**transversetemporal**	596	112	0,093782942	0,01762364	136,7231638
**insula**	3266	2359	0,513917934	0,37119792	32,24888889

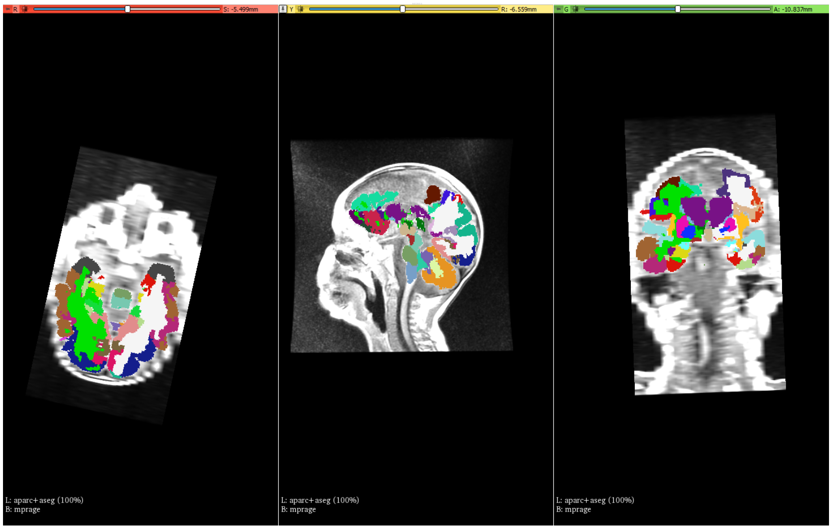

Figure 2: Parcelación de estructuras

Volumetría

Tabla de Colores Lobulos		
1		unknown
2		bank ssts
3		caudal anterior cingulate
4		caudal middle frontal'
5		corpus callosum
6		cuneus
7		entorhinal
8		fusiform
9		inferior parietal
10		inferior temporal
11		isthmuscingulate
12		lateral occipital
13		lateral orbito frontal
14		lingual
15		medial orbito frontal
16		middle temporal
17		parahippocampal
18		paracentral
19		parsopercularis
20		parsorbitalis
21		parstriangularis
22		pericalcarine
23		postcentral
24		posterior cingulate
25		precentral
26		precuneus
27		rostral anterior cingulate
28		rostral middle frontal
29		superior frontal
30		superior parietal
31		superior temporal
32		supramarginal
33		frontal pole
34		temporal pole
35		transverse temporal
36		insula

Figure 3: Tabla de colores lóbulos

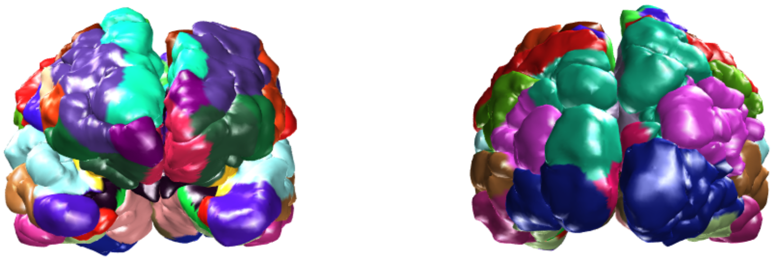

Frontal Posterior

Figure 4: Vista Coronal

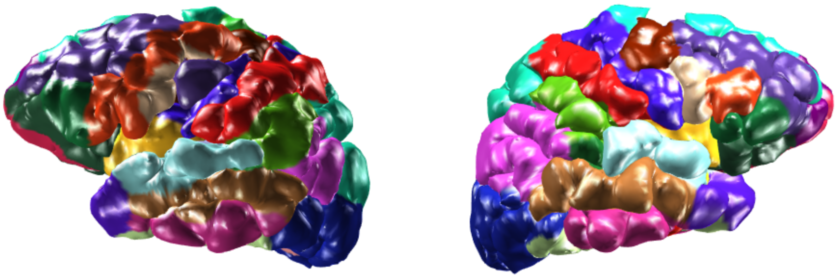

Izquierdo Derecho

Figure 5: Vista Sagital

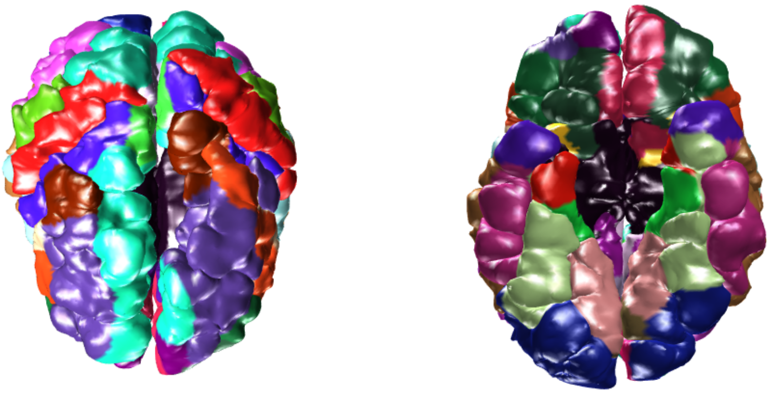

Superior Inferior

Figure 6: Vista Axial

Reporte de Volumetría

Grupo de Investigación en Salud Comfamiliar

Volumen

Table 1: Información Básica

Paciente ID	Sexo	Edad	Fecha Resonancia
1	Masculino	39 Meses	24/04/2018

Table 2: Volumen Macro

Tissue	mm^3	%
White Matter	88111,33023	13,90299288
Gray Matter	201976,8174	31,86970673
Brain	290088,1476	45,77269961
Intracranial Volume	633758,0044	100

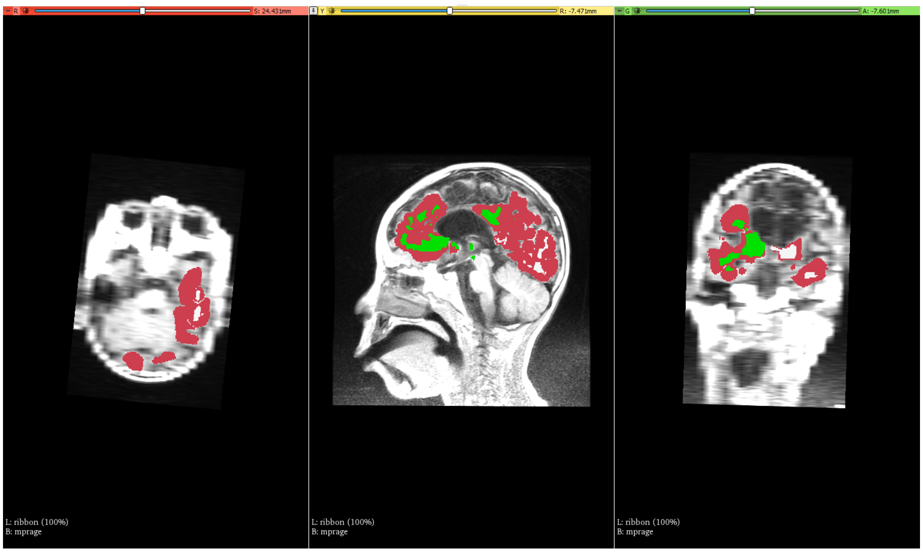

Figure 1: WM y GM

Table 3: Volumen Hemisferios

Tissue	Left (mm^3)	Right (mm^3)	Left (%)	Right (%)	Asymmetry (%)
**White Matter**	34722,56162	53388,76861	5,478835987	8,424156893	42,36959525
**Gray Matter**	87028,17665	114948,6407	13,73208323	18,13762349	27,64719676
**Total**	121750,7383	168337,4093	19,21091922	26,56178039	32,11897586

Table 4: Volumen Estructuras

structure_name	lf_volume (mm^3)	rh_volume (mm^3)	lf_volume (%)	rh_volume (%)	Asymmetry (%)
**fusiform**	3618	10363	0,570880364	1,635166724	96,48809098
**inferiorparietal**	5810	8887	0,916753707	1,402270257	41,87249098
**inferiortemporal**	21192	1092	3,343863092	0,172305516	180,3984922
**lateraloccipital**	3685	62979	0,581452222	9,937389282	177,8891156
**lateralorbitofrontal**	7298	3045	1,151543641	0,480467304	82,23919559
**middletemporal**	1780	13	0,280864303	0,002051256	197,0998327
**postcentral**	3199	2541	0,5047668	0,400941682	22,92682927
**precuneus**	695	20967	0,109663309	3,308360582	187,1664666
**superiorparietal**	29836	5853	4,707790638	0,923538631	134,3999552
**superiortemporal**	1180		0,186190942		
**supramarginal**	504		0,079525623		
**insula**	8473		1,336945639		

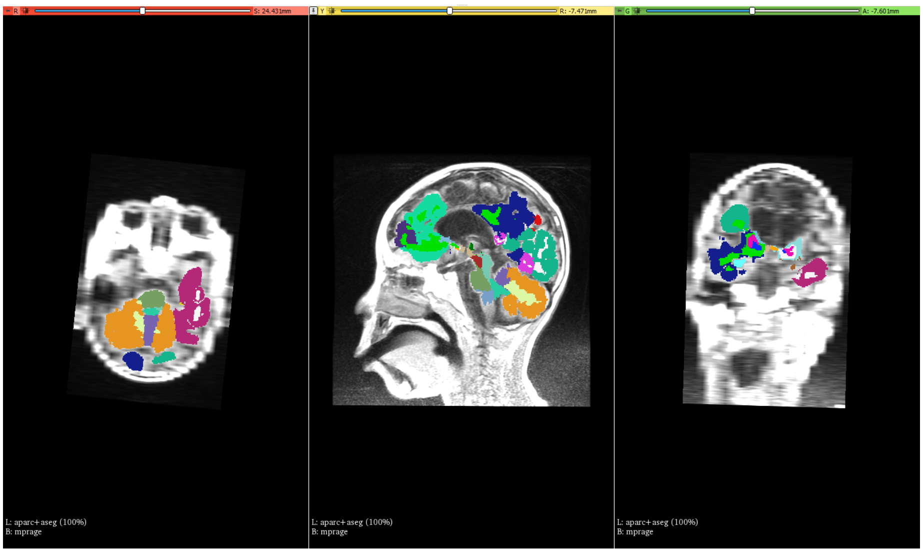

Figure 2: Parcelación de estructuras

Volumetría

Tabla de Colores Lobulos		
1		unknown
2		bank ssts
3		caudal anterior cingulate
4		caudal middle frontal'
5		corpus callosum
6		cuneus
7		entorhinal
8		fusiform
9		inferior parietal
10		inferior temporal
11		isthmuscingulate
12		lateral occipital
13		lateral orbito frontal
14		lingual
15		medial orbito frontal
16		middle temporal
17		parahippocampal
18		paracentral
19		parsopercularis
20		parsorbitalis
21		parstriangularis
22		pericalcarine
23		postcentral
24		posterior cingulate
25		precentral
26		precuneus
27		rostral anterior cingulate
28		rostral middle frontal
29		superior frontal
30		superior parietal
31		superior temporal
32		supramarginal
33		frontal pole
34		temporal pole
35		transverse temporal
36		insula

Figure 3: Tabla de colores lóbulos

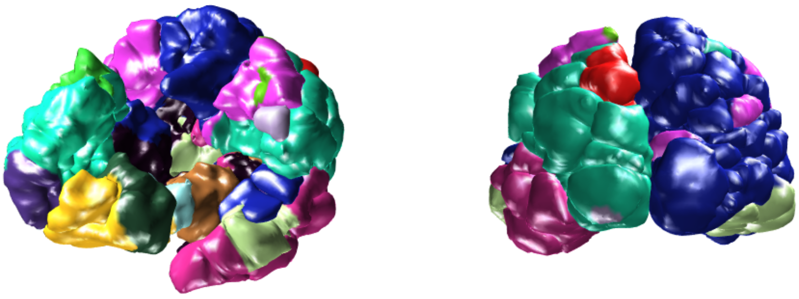

Frontal

Posterior

Figure 4: Vista Coronal

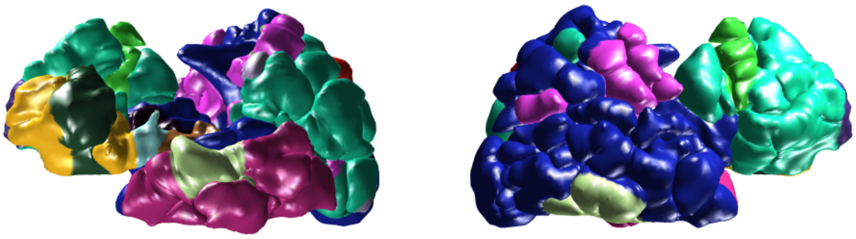

Izquierdo Derecho

Figure 5: Vista Sagital

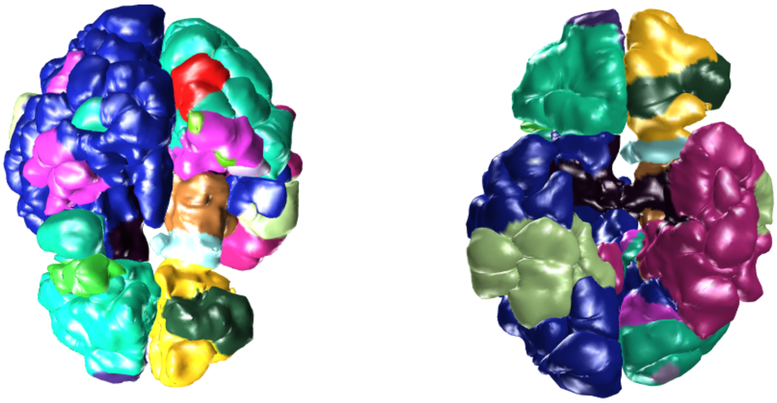

Superior Inferior

Figure 6: Vista Axial

Reporte de Volumetría

Grupo de Investigación en Salud Comfamiliar

Volumen

Table 1: Información Básica

Paciente ID	Sexo	Edad	Fecha Resonancia
1	Masculino	4 Días	31/01/2015

Table 2: Volumen Macro

Tissue	mm3̂	%
**White Matter**	124165,1725	36,04492
**Gray Matter**	159476,389	46,2957
**Brain**	283641,5615	82,34062
**Intracranial Volume**	344473,4489	100

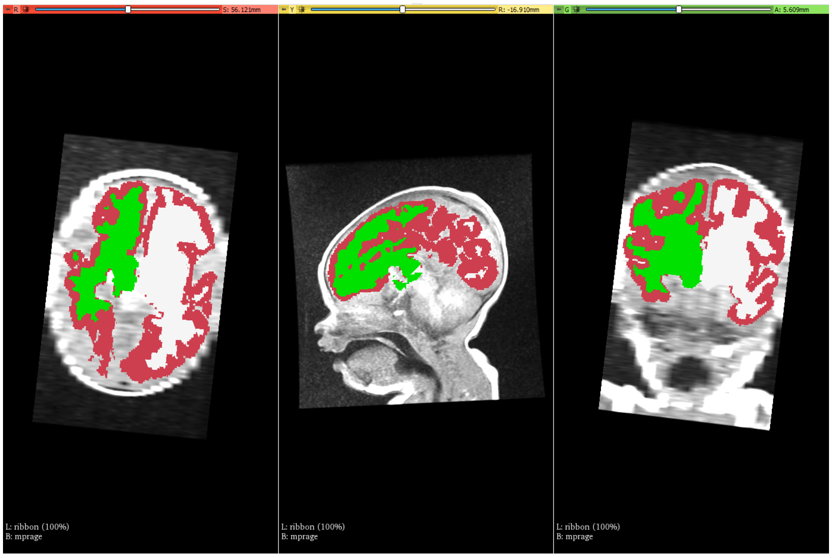

Figure 1: WM y GM

Table 3: Volumen Hemisferios

Tissue	Left (mm$\hat{3}$)	Right (mm$\hat{3}$)	Left (%)	Right (%)	Asymmetry (%)
**White Matter**	64755,89986	59409,27263	18,798517	17,24640109	8,61212065
**Gray Matter**	84093,83031	75382,55866	24,41228216	21,88341624	10,92484186
**Total**	148849,7302	134791,8313	43,21079916	39,12981733	9,912439353

Table 4: Volumen Estructuras

structure_name	lf_volume (mm$\hat{3}$)	rh_volume (mm$\hat{3}$)	lf_volume (%)	rh_volume (%)	Asymmetry (%)
**bankssts**	795	700	0,230787018	0,203208695	12,7090301
**caudalanteriorcingulate**	588	664	0,170695304	0,192757962	12,14057508
**caudalmiddlefrontal**	1487	1749	0,431673328	0,507731439	16,19283066
**cuneus**	773	1809	0,224400459	0,525149327	80,24786987
**entorhinal**	410	462	0,119022236	0,134117739	11,9266055
**fusiform**	2365	1497	0,686555091	0,434576309	44,95080269
**inferiorparietal**	5667	4436	1,645119535	1,28776253	24,36899931
**inferiortemporal**	2481	1809	0,720229675	0,525149327	31,32867133
**isthmuscingulate**	1257	841	0,364904757	0,244140732	39,65681602
**lateraloccipital**	6127	4860	1,778656677	1,410848939	23,06362064
**lateralorbitofrontal**	1387	1489	0,402643514	0,432253924	7,093184979
**lingual**	3384	2757	0,982368891	0,80035196	20,42012702
**medialorbitofrontal**	1233	1323	0,357937601	0,384064434	7,042253521
**middletemporal**	2647	2787	0,768419165	0,809060904	5,152741995
**parahippocampal**	553	549	0,160534869	0,159373676	0,725952813
**paracentral**	1677	1908	0,486829974	0,553888843	12,88702929
**parsopercularis**	1116	1005	0,323972719	0,291749626	10,46676096
**parsorbitalis**	415	560	0,120473726	0,162566956	29,74358974
**parstriangularis**	1098	1103	0,318747353	0,320198844	0,454338937
**pericalcarine**	1034	1154	0,300168272	0,335004049	10,96892139
**postcentral**	6080	3089	1,765012665	0,896730941	65,24157487
**posteriorcingulate**	994	1004	0,288556347	0,291459328	1,001001001
**precentral**	4381	5138	1,271796132	1,491551821	15,90503204
**precuneus**	3412	4036	0,990497239	1,171643276	16,75617615
**rostralanteriorcingulate**	505	343	0,146600559	0,099572261	38,20754717
**rostralmiddlefrontal**	3659	3789	1,062200878	1,099939636	3,490870032
**superiorfrontal**	5642	6083	1,637862082	1,765883559	7,52238806
**superiorparietal**	8361	7435	2,427182712	2,158366639	11,72448721
**superiortemporal**	4950	3621	1,436975772	1,051169549	31,01155058
**supramarginal**	5967	3765	1,732208976	1,092972481	45,25277435
**frontalpole**	182	597	0,052834261	0,173307987	106,5468549
**temporalpole**	786	992	0,228174335	0,287975751	23,17210349
**transversetemporal**	401	339	0,116409552	0,098411068	16,75675676
**insula**	2082	1695	0,604400718	0,49205534	20,49245433

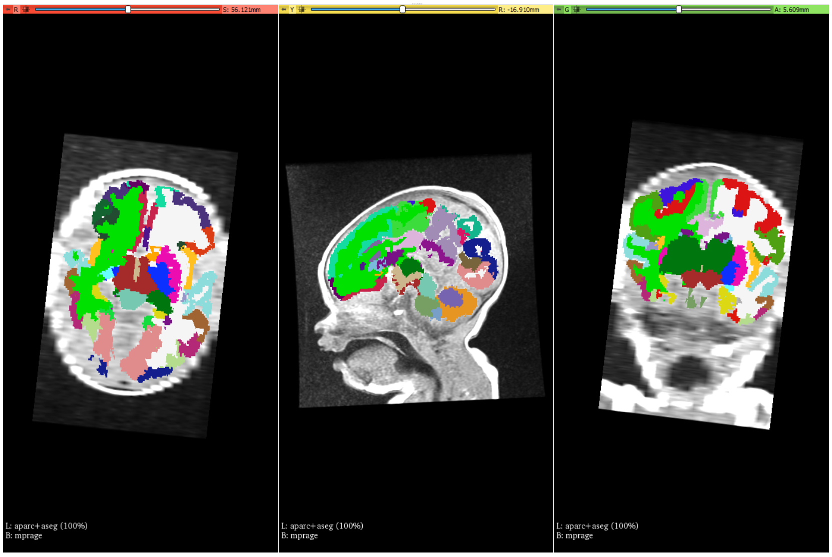

Figure 2: Parcelación de estructuras

Volumetría

Tabla de Colores Lobulos		
1		unknown
2		bank ssts
3		caudal anterior cingulate
4		caudal middle frontal'
5		corpus callosum
6		cuneus
7		entorhinal
8		fusiform
9		inferior parietal
10		inferior temporal
11		isthmuscingulate
12		lateral occipital
13		lateral orbito frontal
14		lingual
15		medial orbito frontal
16		middle temporal
17		parahippocampal
18		paracentral
19		parsopercularis
20		parsorbitalis
21		parstriangularis
22		pericalcarine
23		postcentral
24		posterior cingulate
25		precentral
26		precuneus
27		rostral anterior cingulate
28		rostral middle frontal
29		superior frontal
30		superior parietal
31		superior temporal
32		supramarginal
33		frontal pole
34		temporal pole
35		transverse temporal
36		insula

Figure 3: Tabla de colores lóbulos

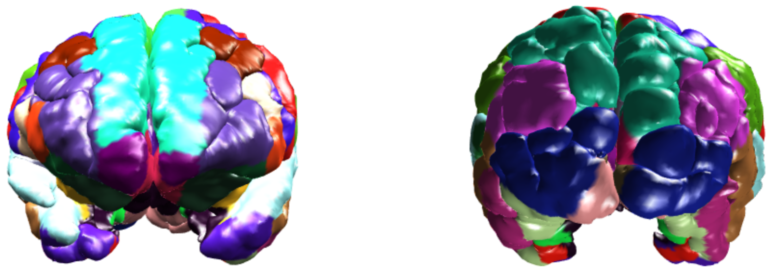

Frontal Posterior

Figure 4: Vista Coronal

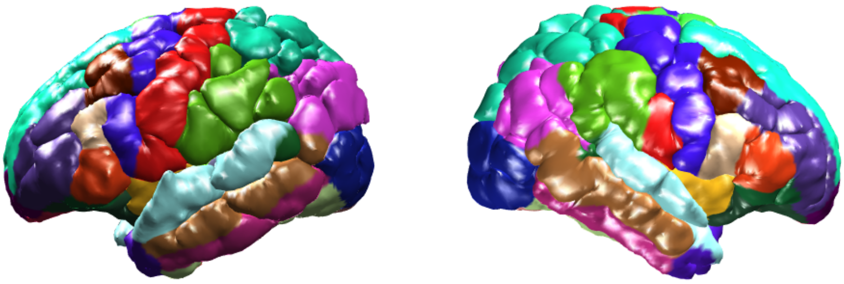

Izquierdo Derecho

Figure 5: Vista Sagital

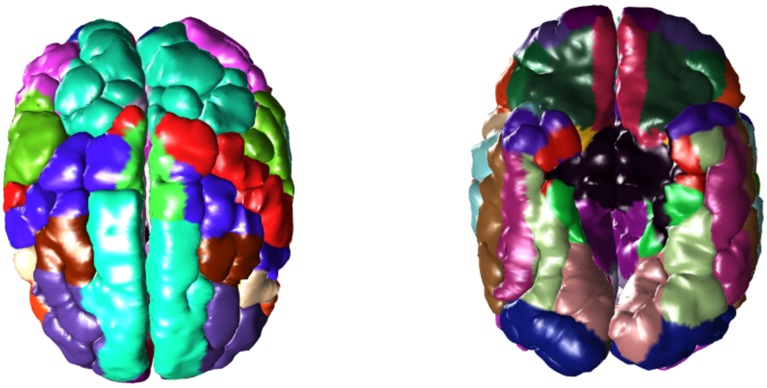

Superior Inferior

Figure 6: Vista Axial
